# Evaluation of *MFRP* as a candidate gene for high hyperopia

**Published:** 2009-01-23

**Authors:** Panfeng Wang, Zhikuan Yang, Shiqiang Li, Xueshan Xiao, Xiangming Guo, Qingjiong Zhang

**Affiliations:** State Key Laboratory of Ophthalmology, Zhongshan Ophthalmic Center, Sun Yat-sen University, Guangzhou, China

## Abstract

**Purpose:**

Mutations in the membrane-type frizzled-related protein (*MFRP*) gene have been identified in patients with pathologic high hyperopia associated with nanophthalmos or microphthalmia. This study is to test if a mutation in *MFRP* is responsible for physiologic high hyperopia.

**Methods:**

DNA was prepared from venous leukocytes of 51 patients with physiologic high hyperopia (refraction of spherical equivalent ≥+5.00 [diopters] D) and 96 controls (refraction of spherical equivalent between −0.50 D and +1.00 D). The coding regions and adjacent intronic sequence of *MFRP* were amplified by polymerase chain reaction (PCR) and were then analyzed by cycle sequencing. Variations detected were further evaluated in normal controls and available family members by heteroduplex- single-strand conformation polymorphism (SSCP) analysis or sequencing.

**Results:**

The average spherical refractive error of patients was +8.41 D in the right eye (from +6.00 D to +16.5 D) and was +8.76 D in the left eye (from +6.00 D to +16.5 D). Five novel heterozygous variations in *MFRP*, c.55-14_55-13insGTAT, c.496C>G, c.664C>A, c.669G>A, and c.770G>A, were identified. Of these, c.664C>A (p.Pro222Thr) and c.669G>A (p.=) were not observed in the 96 normal controls. In addition, one known c.192C>G substitution and five single nucleotide polymorphisms (SNPs; rs883247, rs3814762, rs36015759, rs2510143, and rs35885438) were detected.

**Conclusions:**

Several novel variations in *MFRP* were detected in Chinese. Our results imply that *MFRP* is less likely to play a major role in physiologic high hyperopia.

## Introduction

Hyperopia, alternatively termed hypermetropia or farsightedness, is a common refractive error in children and adults [1-3]. Hyperopia may be classified as low hyperopia (+2.00 diopters [D] or less), moderate hyperopia (between +2.25 D and +5.00 D), and high hyperopia (over +5.00 D) [4]. The prevalence of hyperopia (+3 D or higher) in adults is 9.9%, 11.6%, and 5.8% in the USA, Western European nations, and Australia, respectively [5]. Patients with high hyperopia may suffer from blurred vision, asthenopia, accommodative dysfunction, binocular dysfunction, amblyopia, strabismus, closure angle glaucoma, and retinal detachment. The morphological characteristics of high hyperopia include a short eye axis, a narrow angle between the cornea and iris, expansion of the choroidal vascular bed, and a thickening of sclera connective tissue [6]. Most cases of high hyperopia are physiologic high hyperopia that is not associated with other ocular or systemic anomalies. However, pathologic high hyperopia may present as a part of other eye abnormalities such as nanophthalmos, microphthalmia, anterior segment malformations, and Leber congenital amaurosis [7-9] while there are also numbers of high hyperopia associated with systemic syndromes such as Down syndrome and fragile X syndrome [10,11]. Compared with myopia, hereditary factors may play a more important role than environmental factors in the development of high hyperopia [12,13]. Mutations in *MFRP* have been identified to be responsible for nanophthalmos 2 (NNO2, OMIM 609549) and microphthalmia (OMIM 611040), where extremely high hyperopia (+8.00 D-+25.00 D) is a prominent sign [6,8,14]. However, whether variations in *MFRP* play any role in physiologic high hyperopia is still unclear, although *MFRP* in 11 individuals with hyperopia (4 of the 11 with high hyperopia) has been analyzed [14].

The membrane-type frizzled related protein gene (*MFRP*; OMIM 606227), located in 11q23, is comprised of 13 exons, which translate into a 579 amino acid protein. Selectively expressed in the retinal pigment epithelium and the ciliary body with C1q and tumor necrosis factor related protein 5 (CTRP5) [6,15], the protein contains three domains, a cysteine-rich domain (CRD) at the COOH-terminus, a type II transmembrane domain, and an extracellular region with two tandem-repeats of cubilin (CUB) at the NH_2_-terminus [16]. The CRD can bind with Wnts (wingless type proteins), which belong to a family of cell signaling molecules that are possibly involved in eye development through mediating cell growth [17]. Additionally, CUB is homologous with the low density lipoprotein (LDL) receptor. Normal function of *MFRP* is essential for the eye to reach its full size at birth and is necessary for emmetropization, which is associated with the regulation of ocular axial growth [18]. Although a splice mutation of *Mfrp* causes a recessive retinal degeneration in the rd6 mouse [19,20], recessive mutations of *MFRP* have been detected in humans with nanophthalmos [8] and have been reported to be associated with an autosomal recessive ophthalmic syndrome characterized by posterior microphthalmos, retinitis pigmentosa, foveoschisis, and optic disc drusen [21,22]. Since the simple hyperopia cases represent a less severe form of a short axial length that is similar to the delineation of nanophthalmos [18], *MFRP* may also be the etiological factor of simple high hyperopia. The identification of the causative or susceptibility genetic factors involved in simple high hyperopia will have clinical implications for both ophthalmology and optometry.

Here, *MFRP* was analyzed in 51 Chinese patients with physiologic high hyperopia to determine whether *MFRP* plays a role in the development of high hyperopia.

## Methods

### Patients and clinical data

A previously established and published procedure was used for collecting subjects and obtaining informed consent [23]. This study followed the tenets of the Declaration of Helsinki and was approved by the Institutional Review Board of Zhongshan Ophthalmic Center (Guangzhou, China). Chinese subjects with high hyperopia who met the following criteria were recruited: 1) they had bilateral refraction errors of +5.00 D or higher (spherical equivalent) and 2) they had no other known ocular or systemic diseases. A group of 96 controls met the following criteria: 1) bilateral refraction between –0.50 D and +1.00 D and 2) no family history of hyperopia. The refractive error for all eyes was measured with cycloplegic autorefraction after mydriasis (Mydrin®-P, a compound tropicamide; Santen Pharmaceutical Co. Ltd., Osaka, Japan). Ophthalmologic examinations were performed by ophthalmologists (Z.Y., Q.Z., and X.G.). Genomic DNA was prepared from the venous blood of 51 unrelated patients with high hyperopia and of the 96 normal controls.

### Variation analysis

Eight pairs of primers were used to amplify the 13 coding exons and the adjacent intronic sequence of *MFRP* (human genome build 36.2 NC_000011.8 for gDNA, NM_031433.1 for cDNA, and NP_113621.1 for protein; Table 1). The polymerase chain reaction (PCR) products for the individual exons from each patient were sequenced with the ABI BigDye Terminator cycle sequencing kit version 3.1 (Applied Biosystems, Foster City, CA) according to the manufacturer's recommendations using an ABI 3100 Genetic Analyzer (Applied Biosystems). Sequencing results from the patients as well as *MFRP* consensus sequences from the NCBI human genome database (NM_031433.1) were imported into the SeqManII program of the Lasergene package (DNASTAR Inc., Madison, Wisconsin) and aligned to identify variations. Each variation was confirmed by bidirectional sequencing. The nomenclature of the variation was performed according to the directions recommended by the Human Genomic Variation Society. Any detected novel variation in *MFRP* was further evaluated by either heteroduplex-SSCP (single-strand conformation polymorphism) analysis [24] or by direct sequencing in available family members as well as in the 96 normal controls. Extra pairs of primers were designed for heteroduplex-SSCP analysis (Table 2). Five variations (rs36015759, rs2510143, c.55-14_55-13insGTAT, c.496C>G, and c.770G>A) were genotyped and tested for association using Fisher’s exact test with a significance level threshold of 0.05.

**Table 1:** Primers used for polymerase chain reaction amplification and sequencing of *MFRP*.

**Exon**	**Primer sequence (5'-3')**	**Size of PCR product (bp)**	**Annealing temperature (°C)**	**Note**
1,2	F-CTTTTCCCTCGGGTAGTAGA	466	60	GC buffer
	R-GTTGGCAGGTGGGGTTTTGAA			
3,4	F-AGGACAGCATGAGGAATACC	504	62	
	R-AACCCCACCCCGTCATCTTG			
5	F-GAGAAGGGCCCAAGATGACG	405	63	GC buffer
	R-TCCCTGCCACTCCCTGATTC			
6,7	F-TTGGGGGTTGAGAAAATAGG	601	58	
	R-CTGGGCCAAAGAATGACTGA			
8	F-ATCACCGCCAGCCCTATTG	281	62	
	R-CATCCCCCGTCTGCTTGAT			
9	F-GGTGCCCGGGATGAGACAG	267	62	GC buffer
	R-CCGGGGGTGGCAGACAGT			
10,11	F-GCAGTGCCCCCTCAGTCAGC	511	64	
	R-GTGGGCACCCAGCCTGCTC			
12,13	F-CAGGCCACAGAGCCAGTGAG	548	63	
	R-AGCCCTGACCGGCAAAAGAG			

GC buffer was provided by Takara Biotechnology (Dalian) CO.Ltd (Liaoning,China) and designed for amplification of templates having complex secondary structure or high GC content. In the "Primer sequence" column, "F" indicates the forward sequence and "R" indicates the reverse sequence.

**Table 2:** Primers used for evaluating the variations by heteroduplex-single strand conformational polymorphism analysis.

**Variations**	**Primer sequence (5'-3')**	**Size of PCR product (bp)**	**Annealing temp (°C)**
c.192C>G	F-TGCCCTCAGCCCTCAAGTATC	340	66
	R-CAGCCCAAGCAGCAGGAGGAG		
c.55-14_55-13insGTAT	F-CTTTTCCCTCGGGTAGTAGA	344	62
	R-GACGCTGTAGCTGGCATCCT		
c.664C>A	F-TCTCTGGCTGACCCTGCTCTT	152	60
c.669G>A	R-AAACCAAATCCTTCCACACTGCT		
c.770G>A	F-TTGGGGGTTGAGAAAATAGG	283	60
	R-TGGGTGGAGGGGAAGAAAGTG		

In the "Primer sequence" column, "F" indicates the forward sequence and "R" indicates the reverse sequence.

## Results

**Figure 1:**
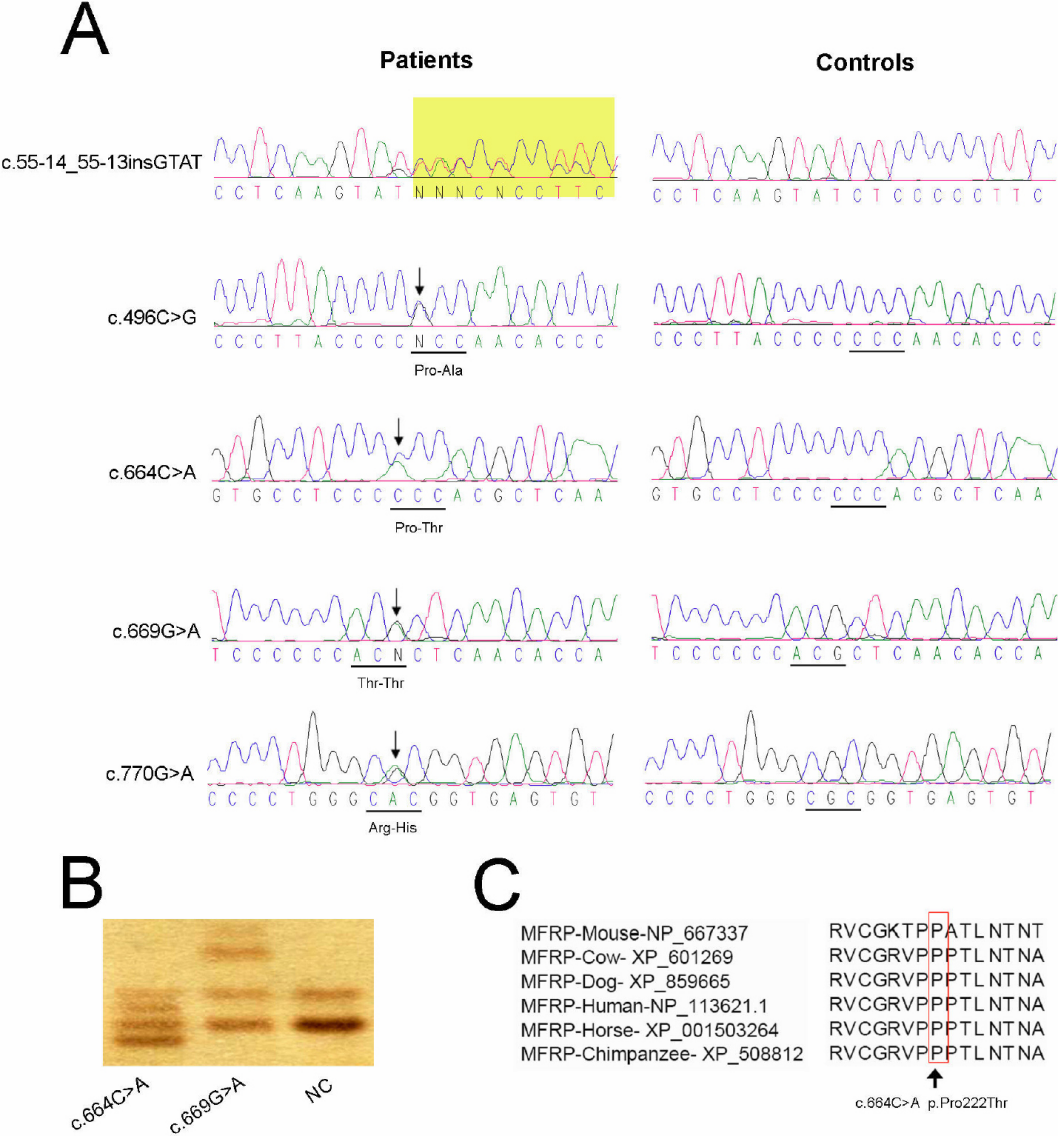
The five novel variations identified in *MFRP*. **A**: The sequences of variations (indicated by an arrow) are shown on the left to compare with the wild type sequences on the right. **B**: The variations, c.664C>A and c.669G>A, were not observed in normal controls (NC) by heteroduplex-SSCP analysis. **C**: The mutation, c.664C>A (indicated by an arrow), changed a highly conserved residue from proline to threonine.

A total of 51 unrelated patients with high hyperopia were recruited into our study. The average spherical refractive error of patients was +8.41 diopters in the right eye (from +6.00 D to +16.5 D) and was +8.76D in left eye (form +6.00D to +16.5 D). Direct sequencing identified 11 heterozygous variations in *MFRP* where the following five variations were novel: c.55-14_55-13insGTAT, c.496C>G, c.664C>A, c.669G>A, and c.770G>A (Table 3 and Figure 1A). Except for c.664C>A and c.669G>A, the rest of the novel variations were observed in the normal controls by heteroduplex-SSCP analysis (Figure 1B). For the missense variation, c.664C>A (p.Pro222Thr), which was identified in one patient, the threonine (a small, hydrophobic amino acid carrying charge) took the place of proline (a small amino acid), which resulted in a change at the protein level with a residue weight of −1 (evaluated by amino acid substitution matrices Blosum 62). The proline at position 222 is highly conserved for MFRP as demonstrated by an analysis of six orthologs from different vertebrate species (Figure 1C). For the synonymous variation, c.669G>A, observed in two patients, the substitution did not change the amino acid or transcript splicing. In addition, a reported substitution, c.192C>G [25], and five SNPs (rs883247, rs3814762, rs36015759, rs2510143, and rs35885438) were observed. No statistical association was observed for variations (rs36015759, rs2510143, c.55-14_55-13insGTAT, c.496C>G, and c.770G>A) between high hyperopes and controls.

**Table 3:** Sequence variations in *MFRP* observed in 51 patients with hyperopia.

**Variations**	**Location**	**Change**	**Effect**	**Hyperopia (n=51)**	**Normal control (n=96)**
c.1–31G>A	5′	GGA-GAA	rs883247	26	ND
**c.55-14_55-13insGTAT**	**Intron 1**	**insertGTAT**	**No effect**	**3**	7*
c.192C>G	Exon 3	CGC-CGG	Arg-Arg	1	0
c.406G>A	Exon 4	GTG-ATG	rs3814762	20	ND
c.492C>T	Exon 5	TAC-TAT	rs36015759	20	36*
**c.496C>G**	**Exon 5**	**CCC-GCC**	**Pro-Ala**	**1**	3*
c.540T>C	Exon 5	CAT-CAC	rs2510143	51	95*
**c.664C>A**	**Exon 6**	**CCC-ACC**	**Pro-Thr**	**1**	**0**
**c.669G>A**	**Exon 6**	**ACG-ACA**	**Thr-Thr**	**2**	**0**
**c.770G>A**	**Exon 6**	**CGC-CAC**	**Arg-His**	**1**	2*
c.954G>A	Exon 8	CTG-CTA	rs35885438	1	ND

The novel variations are highlighted in bold. An asterisk indicates that no statistical difference was observed between patients and controls. ND: no detected.

## Discussion

Recently, *MFRP* was evaluated among 11 hyperopia patients. Five novel variations were identified, but direct sequencing and family-based association analysis of these variations did not implicate *MFRP* in the process of moderate to high hyperopia [14]. We screened this gene in a larger cohort of physiologic high hyperopia and identified five novel sequence variations of *MFRP* from 51 patients. Three of these (c.55-14_55-13insGTAT, c.496C>G, and c.770G>A) appeared to be common *MFRP* polymorphisms because all of them were screened not only in patients but also in normal controls. Two variations, c.664C>A (missense) and c.669G>A (silent), were detected in only patients with high hyperopia.

*MFRP* has already been screened in patients with different kinds of inherited eye diseases such as nanophthalmos 2 (NNO2, OMIM 609549) [26], posterior microphthalmia with retinitis pigmentosa, foveoschisis and optic disc drusen (OMIM 611040) [21,22], retinopathy [27], glaucoma [25,28], and ocular disease related to axial length [14]. Besides six MFRP mutations identified in patients with NNO2 or posterior microphthalmia with retinal degeneration, many non-pathological variations were observed in *MFRP*. Among the six causative *MFRP* mutations, four of them were homozygous (c.498insC, c.498delC, c.524T>C, and c.1143insC), and two of them were compound heterozygous (c.492delC and c.545C>T). Five of the mutations were located in exon 5 (428–641 bp of the consensus coding sequence), which led to an assumption that the tract of short peptide in this region might play a role in the etiology of eye anomalies [21]. We identified a heterozygous missense variation, c.496C>G (p.Pro166Ala), but this variation was also observed in normal controls and the clinically normal mother of the patient with the variation. Although it is less possible for the c.496C>G variation of *MFRP* to account for hyperopia, the recognition of this missense variation in the short tract of exon 5 in *MFRP* will help further the understanding of the role of this area in the function of MFRP.

A silent variation (c.669G>A) presented in *MFRP* of two patients with high hyperopia, but none of the controls carried this change. Although the substitution did not change the encoded amino acid or splice method, it might affect the in vivo stability of the mRNA or protein folding, which is what has been observed for other silent variations in other systems [29-31]. Therefore, it is a variation in *MFRP* that has the potential to cause hyperopia. The missense variation, c.664C>A, resulted in a significant change at the amino acid level as evaluated by Blosum 62 (−1), and this substitution was not identified in the normal controls. Unfortunately, no other family members of this patient were available for us to perform a cosegregation analysis on this variation. More research is needed to reveal the effect of the two *MFRP* variations on simple high hyperopia.

In conclusion, five novel sequence variations of *MFRP* were identified in the Chinese population where two of these novel variations were only present in patients with physiologic high hyperopia. Our results imply that *MFRP* is not likely to play a major role in physiologic high hyperopia. Further studies are needed to clarify this point.
